# The pioneering role of metal–organic framework-5 in ever-growing contemporary applications – a review

**DOI:** 10.1039/d2ra01505f

**Published:** 2022-05-12

**Authors:** Kranthi Kumar Gangu, Suresh Maddila, Sreekantha B. Jonnalagadda

**Affiliations:** Department of Chemistry, Vignan's Institute of Information Technology Duvvada Visakhapatnam-530049 India; School of Chemistry & Physics, University of KwaZulu-Natal, Westville Campus Private Bag X54001 Durban 4000 South Africa jonnalagaddas@ukzn.ac.za +27 31 2603091 +27 31 2607325; Department of Chemistry, GITAM Institute of Science, GITAM University Visakhapatnam 530045 Andhra Pradesh India

## Abstract

MOF-5 with a Zn(ii) cluster and terephthalic acid is a distinctive porous material among the metal–organic frameworks (MOFs), with unique physical, chemical and mechanical properties. MOF-5 based composites possess ample applications in modern chemistry. Huge surface area, suitable pore dimensions and scope of tunability make MOF-5 noteworthy in advanced materials. The extensive features of MOF-5 provided an opportunity for researchers to explore atomic/molecular scale materials. Various MOF-5 based composites have been designed with revamped properties appropriate to the application by altering and fabricating MOF-5 *in situ* or using a post-synthetic approach. Surface modification *via* the dispersion and impregnation of active substances into the pores of MOF-5 enhances its applicability. The boundless topologies and morphologies of MOF-5 combined with other chemical entities has provided opportunities in various fields, including catalysis, gas storage and sensors. The present review illuminates the leading role of MOF-5 and its composites in contemporary applications based on the current literature in heterogeneous catalysis, H_2_ and CO_2_ storage and sensors.

## Abstract 2

MOF-5 with a Zn(ii) cluster and terephthalic acid is a distinctive porous material among the metal–organic frameworks (MOFs), with unique physical, chemical and mechanical properties.

## Graphical abstract

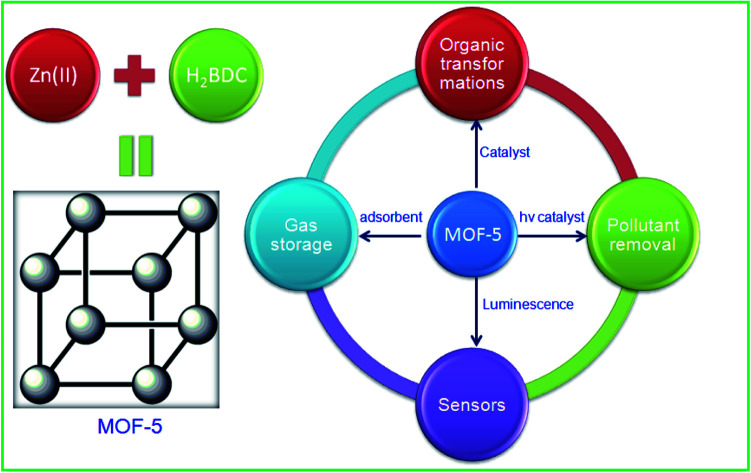

## 1. Introduction

**Fig. 1:**
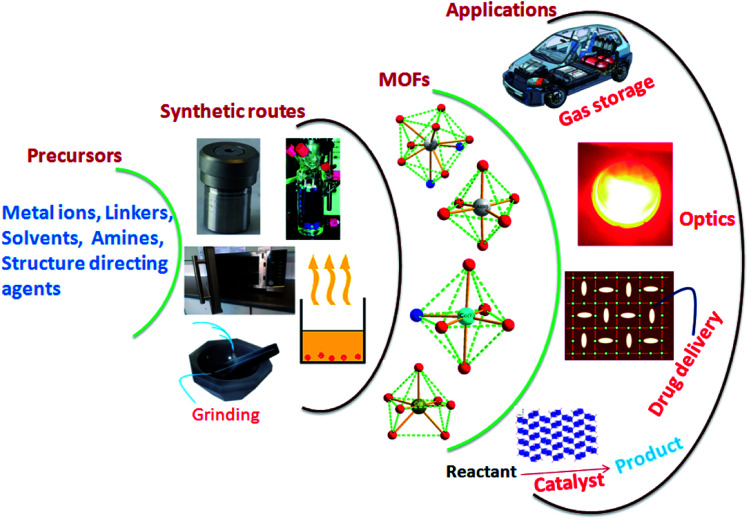
Schematic representation of MOFs synthetic routes and probable applications (this figure has been adapted/reproduced from ref. 27 with permission from Elsevier, copyright 2016).

Metal–Organic Frameworks (MOFs), future prospective advanced materials, arrange metal ions and organic molecules as linkers in various dimensional structures.^1–3^ MOFs are at the leading edge of rapidly emerging materials science. MOFs have attracted researchers from different fields.^4–6^ Characteristics like large surface area, distinctive porous nature, and exceptional structures of choice and rigidity make them ideal for direct use in various applications without further processing.^7–10^ The robust architecture of MOFsis explored in varied fields, including magnetism, luminescence, gas storage, catalysis, and drug delivery, to transform conventional science into technology with added benefits (Fig. 1).^11,12^ The design and construction of MOFs through simple chemical processes is another vital aspect of researchers' interest. Various hydrothermal, sonochemical, microwave-assisted, electrochemical and mechano-chemical synthetic strategies are available for MOFs.The fundamental objective is the bonding between metal ions and linkers with different dimensionalities.^13,14^ The interconnection of entities in the MOFs majorly determines their characteristics. The fabrication of MOFs is a structure–property decisive factor, and its design is generally associated with customised applications. In the MOFs, the inorganic connectors are either isolated metal centres or clusters having different coordination numbers, facilitating the structures of various geometries.^15,16^ Another MOF component, the organic linker, is generally anionic or electrically neutral and sometimes slightly cationic linkers.^17,18^

Rigid ligands are the choice of preference, enabling network geometry and sustaining the open pore structure after removing solvent molecules—a suitable combination of metal centres and linkers is a prerequisite to producing many assemblies.^19–22^ Many MOFs, like MOF-5, MOF-11, MOF-177, MOF-101, MOF-525, and MOF-235, have been reported based on their composition of metal ions and linkers. In designing the MOFs, to improve the properties, some modifications in metal ions linkers are implemented through functionalisation in an *in situ* manner or post-synthetic process.^23–26^

**Fig. 2:**
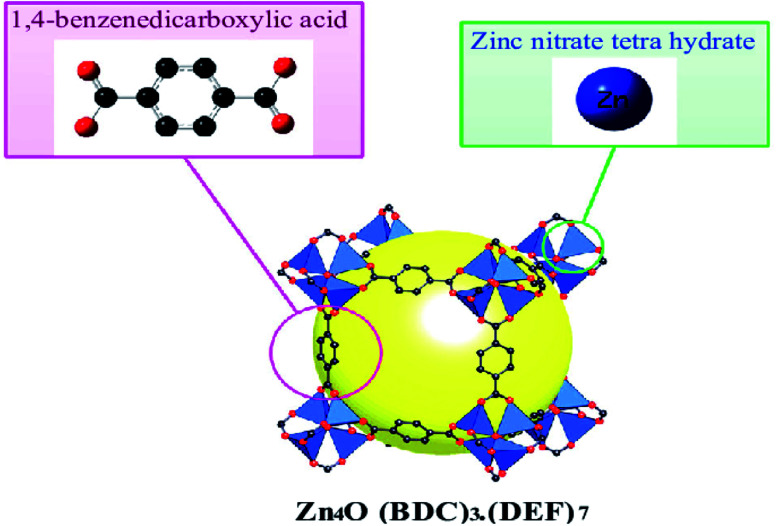
Crystal structures of MOF-5 (C, black; Zn, blue; O, red; gust molecule), yellow sphere and all H atoms omitted for clarity (this figure has been adapted/reproduced from ref. 29 with permission from Longdom Publishing SL, copyright 2014).

MOF-5 is the prominent compound in the MOFs family, constituting the Zn_4_O cluster and 1,4-benzo dicarboxylic acid as an organic linker, first synthesised by the solvothermal technique (Fig. 2). It is a flexible three-dimensional structure with regular internal pore size, facilitating its use in quantum applications. MOF-5 molecules possess a BET surface area from 260 to 4400 m^2^ g^−1^ based on a synthetic method with 0.92–1.04 cm^3^ g^−1^ micropore and thermal stability up to 400 °C. These fascinating parameters help overcome many obstacles encountered with the material used in medicine, biology, *etc.*^27–29^

Utilising the properties of MOF-5, many researchers have explored the material in the catalysis, gas storage and sensor arenas. The design, development, and manufacture of different MOF-5s with new chemical properties, composition, morphology, and topography have broadened its horizon to modern advancements in emerging technologies.^30–32^ Applications in different fields mainly depend on the final structures that possess shape, pore size, and host–guest interactions. Using the same reaction mixture for MOF-5 synthesis can lead to different structures with different characteristic properties. The reaction time, particle size, influence of metal salt, temperature, and morphology generally strongly impact final structure properties like 3D frameworks that exhibit good porosity, making it easy for guest exchange.^33–35^ As mentioned before, the property requirement is paramount in designing MOF-5 and adopting relevant synthetic procedures. Solvents like *N*,*N*′-diethylformamide (DEF), ionic liquids (IL) and structure-directing agents like ammonium ions and amines also influence the final product formation like catenation behaviour of MOF-5. The systematic variation of the amount of water, pH, temperature, time and molar ratio are vital factors in constructing interpenetrated/catenated MOF-5. These variables are applicable to synthesise routes to various MOF-5 morphologies, topologies and composites. The present review encompasses different applications of MOF-5. In most studies, scientists have designed MOF-5 based composites by integrating various engineered materials to improve the desired target application's efficiency, as depicted in Table 1.

**Table 1:** Comparative table of the functionality of MOF-5 and its composites

S. no.	MOF-5/MOF-5 composite	Application	Ref.
1	MOF-5/*n*-Bu_4_NBr (MOF-5 and quaternary ammonium salts)	Cyclic carbonates from CO_2_ and epoxides	40
2	MOF-5-MIX (integration of two different functional groups to ligand)	Conversion of CO_2_ to cyclic carbonates	42
3	Fe(iii)/MOF-5(Ni)	Synthesis of catechol from phenol and hydrogen peroxide	43
4	Nano-sized Rh/MOF-5	Hydroformylation	47
5	Pd/MOF-5	Hydrogenation reaction	48
6	2D MOF-5 using 2-methylimidazole (2-MI) as a coordination controller	Knoevenagel condensation	51
7	NP-C-MOF-5 (nitrogen, phosphorus and porous carbon-doped MOF-5)	Ammonia synthesis	52
8	Nickel-based MOF-5	Oxidation of ethylbenzene (EB)	54
9	PTA@PdCu@Fe^III^–MOF-5 (PTA: Phosphotungstic acid)	Hydrodeoxygenation of palmitic acid to hexadecane	56
10	BiOBr/MOF-5 (IL), (IL: ionic liquid)	Degradation of methyl orange	64
11	MOF-5 on silk fibre	Removal of Congo red from contaminated water	65
12	Ag@MOF-5	*E. Coli* bacteria's disinfection	66
13	HOQ@MOF-5, HOQ: hydroxyquinoline	Visible light-sensitive photocatalyst	69
14	BiOBr/GO/MOF-5, GO: graphene oxide	Photocatalytic activity	70
15	MOF-5 as adsorbent	Tetracycline removal	71
16	MOFMC (MOF-5 integrated with MWCNTs)	Hydrogen storage of 2.02 wt% under 1 bar pressure at 77 K temperatures	79
17	MOF-5	4.5 wt% hydrogen storage at 77 K for 1 bar pressure and 1 wt% at laboratory conditions for 20 bar pressure	90
18	MOF-5	Hydrogen sorption capacities is 1.32 wt% at 77 K and 1 bar pressure	91
19	MOF-5	Hydrogen sorption capacities is 1.6 wt% at 77 K and 1 bar pressure	92
20	MOF-5	Hydrogen uptake is 5.1 wt%at 77 K over 80 bar pressure	93
21	MOF-5	Hydrogen uptake: 2.63 wt% (slow diffusion), 3.2 wt% (direct mixing), and 3.6 wt% (solvothermal) at 77 K and 1.7 MPa	94
22	Meso MOF-5 interpenetrated with TEA	Hydrogen uptake of1.86 wt% under 1 atm at 77 K	98
23	Pt-loaded MWCNTs@MOF-5	Hydrogen uptake:1.25 wt% at ambient temperature over 100 bar pressure and 1.89 wt% at 1 bar pressure at cryogenic temperatures	112
24	Co(ii)-dopedMOF-5	Sensing of solvent molecules	143
25	CH_3_NH_3_PbBr_3_@MOF-5 composite	Fluorescence property	144
26	Ni doped MOF-5	NO_2_detection	145
27	MOF-5	Detection of organophosphate pesticides	146
28	ZnO/MOF-5 hybrid	Enhanced photoluminescence quantum yield of 3.30% compared to bare MOF-5	148
29	Lanthanide ions doped MOF-5	Temperature sensing	151

## 2. MOF-5 and its composites as heterogeneous catalysts for organic transformations

MOFs with exemplary traits have revolutionised the field, and many new composites developed as catalysts for organic transformations, such as Knoevenagel condensation, Heck, Suzuki–Miyaura and Sonogashira reactions.^36,37^ MOFs have profoundly benefited the heterogeneous catalysis arena, as these materials act both as support materials and catalysts due to the unique role of metal ions and ligands. Modified MOF-5sdisplayed salient activity in synthesising many biocompatible drugs.^38,39^

**Fig. 3:**
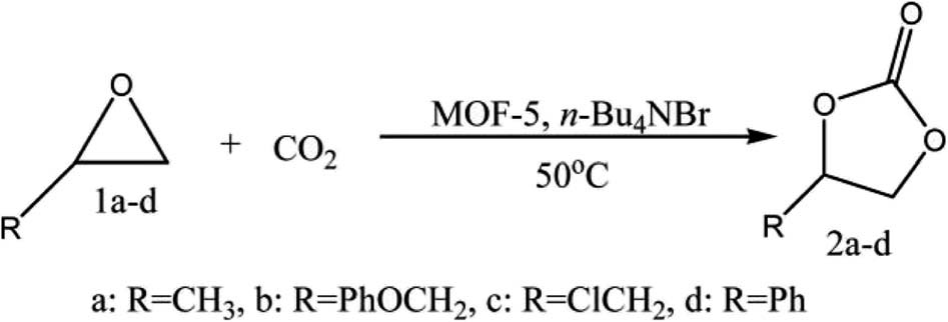
Coupling of CO_2_ with epoxides (this figure has been adapted/reproduced from ref. 41 with permission from Royal Society of Chemistry, copyright 2008).

Jinliang *et al.*^40^ have reported the MOF-5 and quaternary ammonium salt (MOF-5/*n*-Bu_4_NBr) as a catalytic system for synthesising cyclic carbonates from CO_2_ and epoxides. Authors have fused CO_2_ with propylene oxide (PO) in the presence of the catalyst producing propylene carbonate (PC). The inherent catalytic activity of MOF-5 is enhanced by quaternary ammonium salt.^41^ Porous and high surface area MOF-5 becomes accessible to reactants and ammonium salts towards Zn_4_O active sites of MOF-5. Zn_4_O clusters in MOF-5 act as Lewis acidic sites, activating the oxygen atom of epoxides. MOF-5 and ammonium salts' synergetic effect promotes the reaction (Fig. 3), and the absence of a component in MOF-5/*n*-Bu_4_NBr has led to yield depletion. This reaction with MOF-5 as a catalyst is advantageous over other heterogeneous catalysts (metal oxides, functional polymers, gold nanoparticles supported on resins, *etc.*), which demand high-temperature conditions (>100 °C).

A similar coupling reaction was reported by Kurisingal *et al.*^42^ They have prepared multi-variate MOF-5 with functionalised 1,4-benzene dicarboxylic acid with –NH_2_ and –OH groups (MOF-5-MIX). Integration of two different organic ligands into a single framework led to a specialised system with great activity. An advantage is a strategic approach to introducing two functional groups, developing more catalytic active sites than a single functional group. In this study, MOF-5-NH_2_and MOF-5-OH exhibited lower catalytic efficiency in converting CO_2_ to cyclic carbonates than MOF-5-MIX. The Lewis acidic sites (Zn metal sites) and basic sites (functional groups) in MOF-5-MIX are liable to improve catalytic activity.

In another study, Xiang *et al.*^43^ developed a new hydrotable Fe(iii)/MOF-5(Ni) catalyst to synthesise catechol from phenol and hydrogen peroxide. The partial replacement of Zn(ii) ions in MOF-5 with Ni(ii) imparts hydrostability to the structure. [Zn_4_O(BDC)_3_] group in the construction of MOF-5 is likely to interact with a water molecule, which collapses the structure of MOF-5. Thus, Ni(ii) isomorphic to Zn(ii) incorporation improves the hydrophobic nature. The internal pore size of MOF-5 may be accessible and make an easy pass-through of small molecules like phenol and catechol. In this work, initially, Ni(ii) was embedded into the MOF-5 skeleton through *in situ* synthesis followed by Fe(iii) load resulting in Fe(iii)/MOF-5(Ni). High conversion of phenol and high selectivity for catechol were achieved with this catalyst. The drawbacks of phenol conversion (*ca.* 60%) and catechol selectivity (*ca.* 85%) with other catalysts like composite metal oxides, zeolites, organic–metal complexes *etc.*, could be overcome with this Fe(iii)/MOF-5(Ni) catalyst. Phan *et al.*^44^ have used MOF-5 as a solid acid catalyst for the Friedel–Crafts alkylation reaction. Traditionally AlCl_3_, ZnCl_2_, and FeCl_3_ Lewis acids are moisture sensitive, and these catalysts demand dry conditions. MOF-5 is advantageous against leaching problems, frequently encountered in the Friedel–Crafts alkylation with other solid acid catalysts. Moreover, the economic and straightforward synthesis of MOF-5 is beneficial to the chemical industry for utilisation in many organic transformations.^45,46^

Hydroformylation is an essential reaction for the production of aldehydes from olefins. The hydroformylation products are used to synthesise plasticisers, detergents, pharmaceuticals, solvents and agrochemicals. Generally, rhodium complexes are used as a homogeneous catalyst for the hydroformylation process, but their separation from the reaction mixture is a constraint. Vu *et al.*^47^ designed a nano-sized Rh/MOF-5 solid complex to overcome the catalyst recovery problem. The Rh/MOF-5 improved selectivity and performance in olefines' hydroformylation towards aldehydes. Similarly, Opelt *et al.*^48^ have prepared palladium-supported MOF-5 for hydrogenation reaction. Catalyst activity depends on active sites' location and nature, with other catalysts grafted on MOFs' structure. The hydrogenation of ethyl cinnamate to hydro-ethyl cinnamate with 100% conversion and selectivity was reported with Pd/MOF-5. Higher activity with Pd/MOF-5 was due to the excellent dispersion of Pd and increased access on the surface of MOF-5. Another hydrogenation catalyst was developed by Zhao *et al.*^49^ through impregnation of 2–6 nm, Ni dispersed on MOF-5. They used it for the hydrogenation of the C=C bond in crotonaldehyde. After loading nickel nanoparticles, the BET surface area of MOF-5 decreased due to the partial filling of nickel particles in pore channels. The pore volume decrement suggested that reactant and product molecules occupied the space. The MOF-5 retains its structure throughout the reaction. The XRD patterns indicate that other metals like Au, Ag and Pt could be similarly loaded in MOF structures to accomplish different catalytic reactions.^50^ Guo *et al.*^51^ have prepared 2D MOF-5 using 2-methylimidazole (2-MI) as a coordination controller. The unique geometry and the rich surface-area-to-volume ratio of 2D MOF-5 accountably perform better than the conventional bulk MOF-5. The introduction of 2-MI in the synthesis of MOF-5 from its precursors is usually beneficial in enhancing its catalytic activity by creating more Lewis basic sites. This study reveals that 2-MI acts as a base for the deprotonation of the ligand's carboxylic acid. The 2-MI attached to MOF-5 restricts the crystal growth and leads to ultra-thin MOF-5 nanostructures. Using this catalyst, the authors have conducted Knoevenagel condensation using aldehyde and malononitrile and produced a 99% yield at room temperature. The 2-MI assisted synthesis of 2D MOF-5 nanosheets in this study led to the creation of more active sites in MOF-5 to make this material a superior catalyst.

**Fig. 4:**
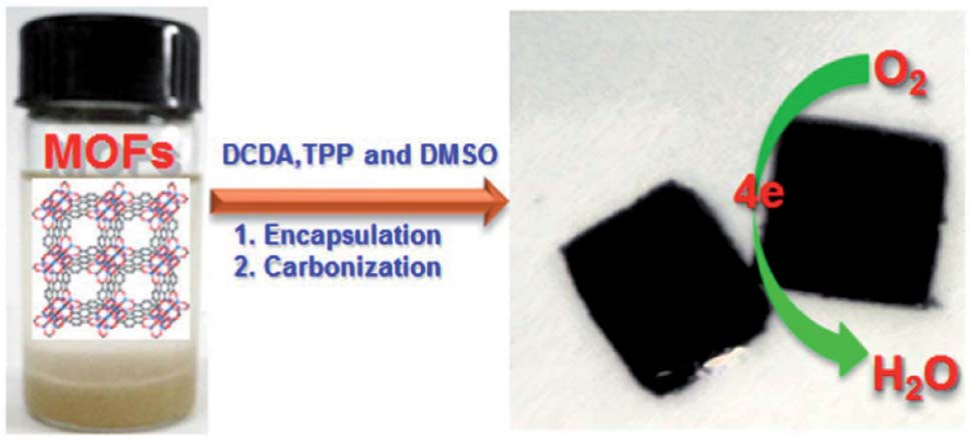
Schematic illustration of the synthesis of MOF-templated NPS-C-MOF-5 as a metal-free electrocatalyst for the ORR (this figure has been adapted/reproduced from ref. 53 with permission from Nature, copyright 2014).

Ammonia synthesis was carried out through nitrogen reduction using NP-C-MOF-5 as an electrocatalyst. Nitrogen, phosphorus and porous carbon-doped MOF-5s were prepared for electrocatalytic nitrogen reduction. Authors have adopted nitrogen reduction reaction (NRR) to synthesise ammonia against the cumbersome Haber–Bosch technique.^52^ Nitrogen-doped carbon has attracted many researchers for electrocatalytic purposes. The intrinsic properties of nitrogen-doped carbons, like high surface area and reasonable pore structure, create electrochemically active sites. The electronic properties like system impedance and surface polarities can be mitigated by including other hetero atoms like phosphorus. MOF-5 was used as support to carry such catalyst to execute NRR and produce ammonia with a yield of 1.08 μg h^−1^ mg_cat_^−1^. Similarly, Li *et al.*^53^ have prepared NPS-C-MOF-5 (Fig. 4). Unlike the above report, sulphur was included and used in the oxygen reduction reaction (ORR). Although the expensive platinum or platinum-based catalysts are favoured, metal-free NPS-C-MOF-5 catalysts exhibit superior properties to commercial platinum-carbon catalysts for ORR. MOF-5 as a template is agreeable for the ternary atoms-doped porous carbons. MOF structure also has a powerful effect on effective ORRspecific MOFs like MOF-5 possess compatible porous structures to accommodate the ternary doped carbon species. Li *et al.* have also studied MOF-5 templated nitrogen and sulphur co-doped porous carbon for ORR. The study described MOF-5 as a friendly electrocatalyst. Its intriguing architecture and comfort to design based on targeted requirements, pore distributions adsorb different organic molecules, and various elements engulfed porous carbons.

**Fig. 5:**
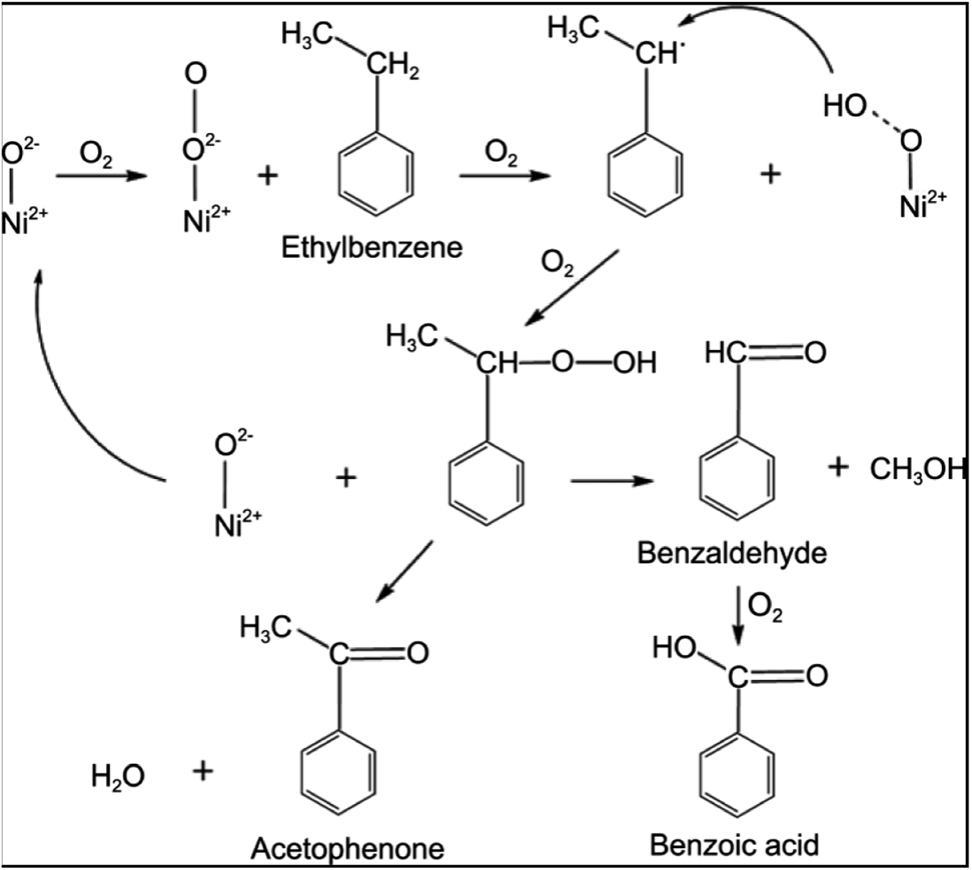
The probable reaction mechanism for EB oxidation over Ni-MOF-5 catalysts (this figure has been adapted/reproduced from ref. 54 with permission from Wiley, copyright 2014).

In another study, Peng *et al.*^54^ prepared Ni nanoparticles supported by MOF-5 using a simple impregnation method. This solid acid catalyst achieved 56% conversion of ethylbenzene (EB) with 90% selectivity towards acetophenone. Nickel-based MOF-5 catalyst for the oxidation of EB like hydrocarbon performed with top results in this study found to be a catalyst of interest in the oxidation of aromatic hydrocarbons. Nickel nanoparticles as NiO species accelerate the conversion of EB in a facile reaction pathway (Fig. 5). Ethylbenzene hydroperoxide intermediate is formed on the catalyst surface and oxidised to form acetophenone and benzoic acid as products.

**Fig. 6:**
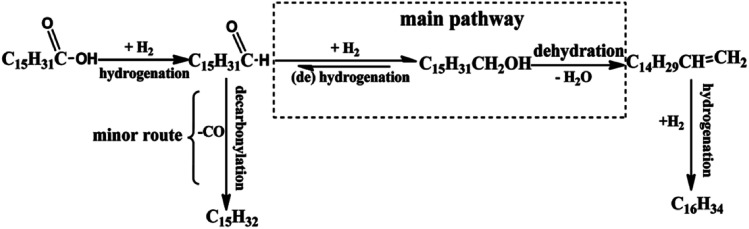
Proposed hydrogenation process for palmitic acid over PTA@PdCu@Fe^III^–MOF-5 (this figure has been adapted/reproduced from ref. 56 with permission from Royal Society of Chemistry, copyright 2017).

Kleist *et al.*^55^ have modified MOF-5 using a mixed linker strategy. 2-Aminobenzene-1,4-dicarboxylate was used as an alternative linker, and functionalisation of the amino group was beneficial to immobilise Pd species. The amino group interaction with Pd leads to its high dispersion and does not block the pores of the MOF-5 structure. Thus, Pd/MIXMOFhas become an effective catalyst in the oxidation of CO at various temperature regions. Using the solvothermal approach, Fang *et al.*^56^ have used MOF-5 support to prepare two catalysts, namely PdCu@Fe^III^–MOF-5 and PTA@PdCu@Fe^III^–MOF-5. Phosphotungstic acid (PTA) encapsulated in the pore structure of MOF-5 of PdCu@Fe^III^–MOF-5 enhanced the acidity. The composite showed improved activity for the hydrodeoxygenation (HDO) of palmitic acid to hexadecane (Fig. 6). The HDO formation over PTA@PdCu@Fe^III^–MOF-5 in a supercritical hexane fluid medium was considered ideal for converting palmitic acid to hexadecane with 99% selectivity.

MOF-5 as support provides exceptional properties compared to polymers (dendrimers, polyaniline), mesoporous solids (mesoporous silica, MCM-41, SBA-16), carbon polymorphs (carbon nanotubes, active carbon, graphene) and metal oxides (Al_2_O_3_, Fe_3_O_4_). In another study, MOF-5 was used to support grafting vibrant metal centres for a copper-free Sonogashira coupling reaction. Palladium supported on MOF-5 exhibited much higher catalytic activity than the other Pd containing support catalysts, as the study reported by Gao *et al.*^57^ It is well-known that Pd without stabilisation renders aggregates and loses catalytic activity. In this study, Pd particles were attached to the surface of MOF-5 instead of into the pores. As per the BET and TEM analysis, Pd nanoparticles size ranged from 3 to 6 nm, but the pore size of MOF-5 ranged between 1 to 2 nm. Thus Pd particles are too large to get into the internal structure of MOF-5. The efficacy of catalysts is distinguishable based on active metal centres' position on substrate molecules. Xiang *et al.*^58^ have prepared Fe(iii)/MOF-5(Ni) catalyst for the synthesis of catechol from phenol and hydrogen peroxide. In this study, the authors have added Ni(ii) into the framework of MOF-5 and partially replaced the Zn(ii) and formed the [Ni_4_O(BDC)_3_], which enhanced the hydrostability of the support. Further, Fe(iii) loaded into MOF-5, and the resulting Fe(iii)/MOF-5(Ni) increased the catalytic activity for the hydroxylation of phenol with hydrogen peroxide to catechol.

## 3. MOF-5 and its composites as photocatalysts in the degradation of pollutants

Population and environment have proved that interlinked subjects and changes in the population invariably impact the environment in many aspects. Many plastics, textiles, pesticides and cosmetics involve toxic substances in the preparation.^59–61^ After their intended use, remnants and metabolites cause toxicity to the environment during the disposal process, even in low concentrations. Removing pollutants is of paramount importance for healthy environment management. Many metal oxides and other semiconducting materials have been explored as adsorbents to remove contaminants. MOFs are unique in this regard, like structure formation, activation, synthesis conditions, pore size, and crystal size, particularly interesting for photocatalysis compared to other counterparts.^62,63^ A few reports have described the role of MOF-5 in photocatalysed reactions.

**Fig. 7:**
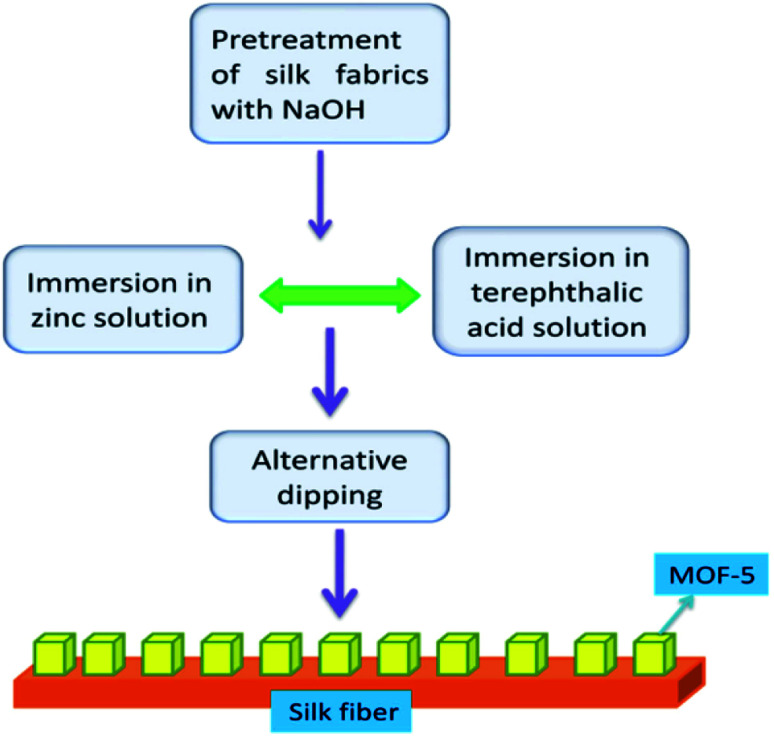
The layer-by-layer deposition of MOF-5 on silk fibre (this figure has been adapted/reproduced from ref. 65 with permission from Elsevier, copyright 2014).

Bismuth oxyhalide (BiOBr) is a visible-light absorbing semiconductor but exhibits limited photocatalytic degradation capability. Lack of effective separation of the photo-generated electrons and holes and deficient surface area for accessing more pollutant target molecules are probable reasons for the low performance of the photocatalytic activity. To alleviate these problems, Yang *et al.*^64^ have prepared MOF-5 *via* the electrochemical method using ionic liquid (IL = 1-butyl-3-methylimidazolium chloride) electrolyte instead of employing conventional solvents like *N*,*N*-dimethylformamide, water/ethanol mixtures or *N*,*N*-diethylformamide. IL induces the porous structure and renders high thermal stability and nonflammability, the desirable properties of the MOF-5 framework structure. BiOBr mixed with MOF-5 (IL) resulted in a composite, and BiOBr/MOF-5(IL) enhanced the photocatalytic capability of BiOBr in the degradation of methyl orange (MO). The degradation ratio, *i.e. C*/*C*_0_ (*C* = current concentration and *C*_0_ = initial concentration), improved to 87.9% when BiOBr was combined with MOF-5 (IL), but using MOF-5 or MOF-5 (IL) singularly, results were not alike. Hence, MOF-5 is a good platform for BiOBr to exhibit enhanced degradation. MOF-5 (IL) increases the accessible surface area and contact capacity of MO molecules with adsorbent. In another study, Khanjani *et al.*^65^ investigated the removal of congo red (CR) from contaminated water with MOF-5. They synthesised MOF-5 on silk fibre by layer-by-layer deposition (Fig. 7). The deposition of MOF-5 on the silk surface showed a high adsorption capacity. MOF-5 with silk fibres opens a vista for various applications, particularly wastewater pollution remediation. The surface area and porosity of MOF-5 have been enhanced with deposition on silk fibre, enhancing photocatalytic activity on successfully removing cargo-red from effluents. The aspartic acid, glutamic acid, and hydroxyl groups present on the silk yarn surface facilitated the facile coatings of MOF-5 on silk fibres.

**Fig. 8:**
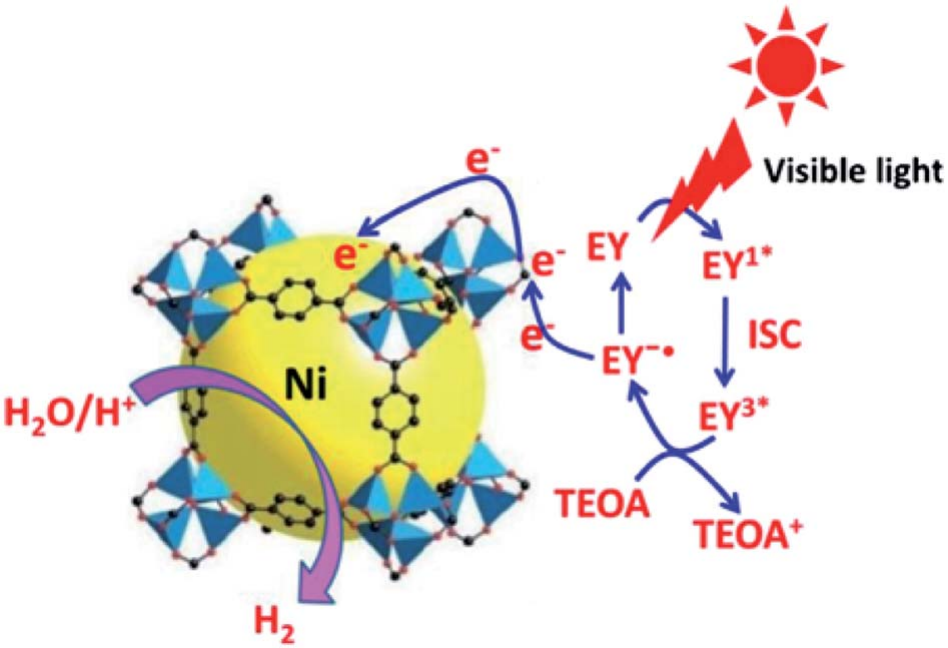
Photocatalytic pathway for H_2_evolution over the EY sensitisedNi@MOF-5 with TEOA under visible light irradiation, EY = Eosin Y (this figure has been adapted/reproduced from ref. 67 with permission from Elsevier, copyright 2016).

Chlorination and ozonation processes can sometimes generate harmful by-products during water treatment. Inexpensive and effective methods are required to inactivate various waterborne pathogens like viruses, fungi, protozoa, cysts, bacteria and algae. Thakare *et al.*^66^ anchored silver nanoparticles on MOF-5 (Ag@MOF-5) and used the material as a representative biological material for the *E. Coli* bacteria's disinfection. Silver nanoparticles are known to possess disinfecting properties. Their incorporation with MOF-5 dramatically enhanced the disinfection properties in the presence of light. The study revealed that oxygen radicals, particularly hydroxyl species, play a vital role in the disinfection of *E. Coli*. Photocatalytic stability of Ag@MOF-5 was active up to seven cycles in the presence of light. At the same time, it showed stable activity up to four times in the absence of light, and then disinfection activity was diminished. Zhen *et al.*^67^ and Thakare *et al.*^68^ modified the basic structure of MOF-5 with small-sized Ni particles and used it for hydrogen generation and in developing visible light active photocatalyst, respectively, as shown in Fig. 8.

Thakare *et al.*,^69^ another study has identified the exceptional advantages of MOFs, particularly the MOF-5 in catalysis science. Its Zn_4_O semiconductor clusters act as light-absorbing antennae and stimulate applications in visible-light photocatalysis. Photocatalysis under visible light is valued as it is inexpensive. TiO_2_ like substances are promising photocatalysts active under ultraviolet light and are less sensitive to the visible spectrum. To prepare a visible light-sensitive photocatalyst, 8-hydroxyquinoline (HOQ) interacted with MOF-5 to synthesise the HOQ@MOF-5 compound. Phenol degradation using the MOF-5 and composite showed that postmodification of MOF-5 enhances its photocatalytic activity. Its robustness is another crucial aspect of practical applications. HOQ@MOF-5 delivers the almost identical photocatalytic efficiency for up to five cycles and can be ideal as a regenerated catalyst. Chen *et al.*^70^ have developed a 2D photocatalytic system, namely BiOBr/GO/MOF-5. BiOBr is a potential photocatalytic semiconductor but needs modification for improved activity. Interfacial compatibility of graphene and unique characteristics of MOF-5 combined with BiOBr improves photocatalytic performance. MOF-5 has provided a high surface area for BiOBr for good scattering, while graphene, because of its good electron mobility, separation of photo-generated electron–hole pairs occurred effectively. Recycled runs up to four times without diminishing photocatalytic activity demonstrate the renewable performance of the MOF-5 composite catalyst. A pharmaceutical product, tetracycline (TC), is usually utilised to treat anthrax, pneumonia, and cholera. The high amounts of expired TC and other antibiotics in waterways are environmental concerns. As per Seyed *et al.*,^71^ TC is effectively removable with MOF-5 as an adsorbent. The operating variables like pH, temperature, initial concentration of TC and adsorbent dosage influenced the adsorption efficiency of MOF-5.

## 4. MOF-5 and its composites as adsorbents for gas uptake

**Fig. 9:**
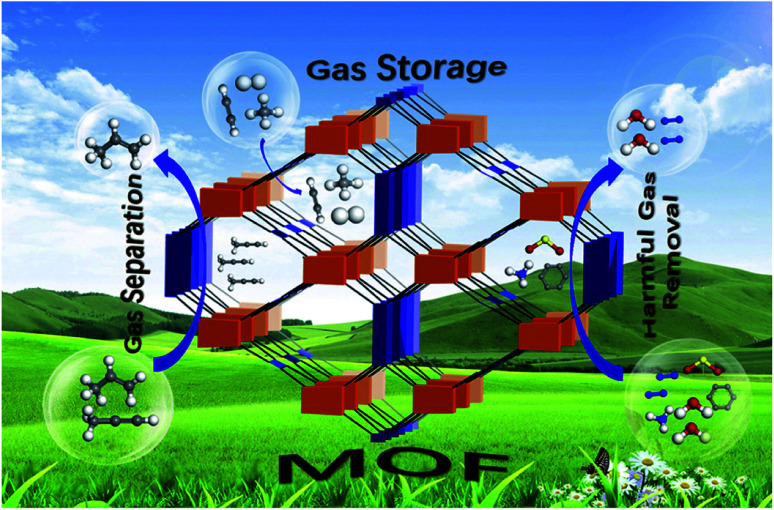
Applications of metal–organic frameworks for green energy and the environment (this figure has been adapted/reproduced from ref. 76 with permission from Elsevier, copyright 2018).

The previous section discussed the effectiveness and environment-friendly nature of MOF-5 composites in eliminating pollutants like pesticides, pharmaceuticals, organic dyes, and pigments from water. However, another vital issue related to environmental sustainability is minimising fossil fuels and finding alternate fuels. Emissions of oxides of carbon, nitrogen, and sulphur are related to global warming and reflect on human health and the eco-system.^72–74^ Hydrogen is eco-friendly fuel and has a tremendous energy density than gasoline, but the major obstacle is the lack of appropriate storage. According to US DOE (US Department of Energy), the onboard efficiency, *i.e.* energy efficiency for delivering hydrogen from the fuel cell's storage compartment, will be 90% by 2025. Besides energy efficiency, hydrogen storage's gravimetric and volumetric capacity has not been achieved by any material as per the DOE targets. Extensive research and investigations are in progress to derive superior methodologies for hydrogen storage. MOF-5 and its composites are on the front line of designing and constructing appropriate storage devices.^75,76^ As in Fig. 9, MOF-5 and its family demonstrate the advances in adsorptive gas separation, storage and removal.

Most recent studies revealed that MOF-5 are non-interpenetrated and have relatively lower storage capacity than interpenetration MOFs at 77 K under 1 bar pressure. The interpenetrated structure improves the performance of MOF-5 to a certain extent. Nevertheless, the complex environment of MOF-5 for the storage or adsorption of hydrogen and ambient moisture instability is a crucial challenge for the broader application of these materials.^77,78^ In this perspective, the hybrid composites (MOFMC) made by integrating MWCNTs treated with acid into MOF-5 demonstrated larger surface areas, enhanced sorption capacities, and enhanced ambient moisture stability. Compared to MOF-5, the hydrogen adsorption isotherms of the interpenetrated hybrid composites (MOFMC) under 1 bar pressure at 77 K showed a higher efficiency. Heng *et al.*^79^ reported that interpenetrated hybrid materials (integrated-MOFMC-mesopore) sorption capacities were greater than MOF-5 and reached 2.02 wt% under 1 bar pressure at 77 K temperatures. Yang *et al.*^80^ conducted similar studies with conventional hybrid materials (MOFMC) and reported 1.52 wt% at the same experimental conditions. It is generally known that interpenetrated MOFs have higher sorption capacity than non-interpenetrated MOFs.^81,82^

Saha *et al.*^83^ reported the adsorption isotherms of crystals obtained from DMF and DEFto store hydrogen at 77 K, 194.5 K, and 298 K, respectively. The uptake capacities of hydrogen for the crystals obtained from DMF and DEF at 1.05 bar pressure and 77 K are 1.2 wt% and 0.7 wt%. The uptake measurements of crystals derived from the DMF are higher than DEF at the remaining temperatures, suggesting that crystals obtained from the DMF have a more remarkable storage ability. The hydrogen uptake capacities on the materials obtained from the DMFdoped with MOF are lesser than MOF-5.^84^ By “physical mixture of the catalyst with MOF-5” and “bridging” (interlinking of MOF-5/catalyst with bridges of carbon), new composites are created. The doped MOF-5, more or less, showed similar hydrogen capacities compared to pristine MOF-5. Thus, the doping of catalysts with MOF-5 did not affect hydrogen enhancement. These composites were related to the surface areas and the hydrogen adsorption isotherms for physisorption. However, they did not show any features related to the spillover effect. Due to the simple synthesis and high stability, the MOF-5s have been more investigated materials among the MOFs.^85–88^ Further, Li *et al.* have reported that in the bridging method, adsorption of hydrogen increases up to 4 wt% for the materials like “IRMOF-8” or 3 wt% for “MOF-5” under similar experimental conditions.^89^

In another study by Liu *et al.*, MOF-5 showed 4.5 wt% hydrogen storage at 77 K for 1 bar pressure and 1 wt% at laboratory conditions for 20 bar pressure.^90^ Rowsell *et al.* have reported hydrogen sorption capacities of MOF-5 are 1.32 wt% at 77 K and 1 bar pressure.^91^ Panella *et al.*^92^ have reported that hydrogen storage capacities of MOF-5 can range 1.6 wt% at the same pressure and temperatures. However, the adsorption capacities of MOF-5 are deficient at the laboratory temperatures, which is less than 0.2 wt% at 67 bar pressure. In his other study, Panella *et al.*^93^ showed that saturation levels of MOF-5 at 77 K over 80 bar pressure for the hydrogen is 5.1 wt% as the combination of MOF-5 is a single crystal. Also, the energy storage by MOF-5 is different, as the different approaches are available for the synthesis. Jinping *et al.*^94^ depict the hydrogen adsorption capacities of MOF-5 materials by three different methods. The adsorption capacities of MOF-5 are 2.63 wt% (slow diffusion), 3.2 wt% (direct mixing), and 3.6 wt% (solvothermal) at 77 K and 1.7 MPa. MOF prepared by the solvothermal process is superior to other methods. The results demonstrate the sample's pore size and areas directly impacting the hydrogen adsorption capacities. Xue *et al.*^95^ developed microporous MOFs (triply interpenetrated) with robust characteristics. These materials yielded a 1.28 wt% hydrogen uptake capacity under 1 bar pressure at −196 °C; thus, about 96% of the overall pore volume was occupied by hydrogen (liquid). Sumida *et al.*^96^ developed a solid MOF with iron consisting of open sites (Fe^2+^) and obtained a weight percentage of 2.3 at 1 bar pressure over −196 °C with adsorption in the heat of nearly 11.9 kJ mol^−1^. Seung *et al.*^97^ reported that MOFs doped with P-, C-, I- and N- showed the hydrogen adsorption capacities of 1.2 wt%, 1.3 wt%, and 1.7 wt%, and 2 wt%, respectively. Even though P-MOF and C-MOF's pore characteristics are analogous, the uptake of hydrogen by P-MOF was greater than by C-MOF; but the uptake capacities of the MOFs reverse at 1 bar pressure. Based on the difference in the pore volumes, smaller than 1 nanometer, the authors justified the variations. Hydrogen storage capacities of the developed structures depend on the network's pore size; the smaller the pore size, the lesser the ability for hydrogen adsorption; thus, facilities with a high pore larger than 1 nm will uptake more hydrogen even at low pressures. At 1 bar pressure, MOFs having a larger pore size than 1 nm influenced the uptake capacities of hydrogen; thus, C-MOF having a larger pore size exceeded the storage capacities of P-MOF.

Feng *et al.*, 2013 (ref. 98) have developed a MOF-5 (interpenetrated) with TEA with high mesoporosity. The study revealed that these materials stored 1.86 wt% of hydrogen under 1 atm at 77 K, more elevated than MOF-5 (interpenetrated) under the same condition. The interpenetrated mesopore and intercrystalline structure of MOF-5 enable hydrogen sorption at even lower pressures. A 1.86 wt% hydrogen adsorption was attained at 1 atm pressure and 77 K, higher than with MOF-5 (interpenetrated) under similar conditions with or without mesopores.^99,100^ Mesopores and macropores of the MOF-5 might be disadvantageous to the sorption. There is no attraction between pore surfaces and molecules of hydrogen near the pore centre; however, the uptake capacities by MOF-5 reached a wt% of 1.86. The results reveal that due to the strong physisorption by unsaturated metal sites, even at low-pressure, intercrystalline mesopores of MOF-5 help the hydrogen uptake.^101^ Thus, hydrogen uptake can be enhanced by the interpenetrated MOFs at low pressures. Therefore, MOF-5 prepared at low pressures, the mesopores with intercrystalline and interpenetration might increase hydrogen sorption.

Kim *et al.*, in another study, developed a composite MOF (C.B./Pt/MOF-5); these are coated with the Zn_4_O (benzene dicarboxylate)_3_ and carbon black (hydrophobic microporous).^102^ The newly created composite uptake capacities reached 0.62%. The pristine MOF is only 0.44%. Thus, it is evident that the composite showed superiority overnew MOF by 42% and showed excellent hydrophobicity at room temperatures. The results indicate that MOF-5 synthesised using platinum nanoparticles and carbon black enhanced hydrogen storage capacities and hydro-stability. For instance, MOF-5 prepared with a zinc cluster unified by the BDC (1,4-carboxylate) showed a sorption capacity of wt% of 1.25 at 77 K and wt% of 0.4 over room temperatures.

Hydrophobic materials are valuable to improve the hydro-stability of MOFs. For instance, Yang *et al.*, 2011 used “methyl groups” (hydrophobic) on moieties of BDC to enhance stability.^103^ Yang *et al.* have developed a MOF-5 composite with hydrophobic carbon nanotubes.^104^ Nguyen *et al.*^105^ have developed ambient moisture resilience MOF by modifying MOFs after post-synthesis. The present technique is useable for the MOFs, which do not require pre-functionalisation. The MOF-5 showed an uptake of wt% 0.44, which is similar to the reports of MOF-5.^106–108^ The MOF-5 loaded with platinum achieved the highest sorption capacities as they are smaller in size and produced higher dispersion of metal (Pt) nanoparticles and resulting in the development of “Pt-hydride” during the spillover mechanism.^109–111^ Seung *et al.*^112^ have reported the synthesis techniques and preparation process of a hybrid composite Pt-loaded MWCNTs@MOF-5 [Zn_4_O(benzene-1,4-dicarboxylate)_3_] loaded with the platinum nanoparticles, and these materials enhanced the uptake capacities at laboratory temperatures. The composite has shown uptake of 1.25 wt% at ambient temperature over 100 bar pressure and 1.89 wt% at 1 bar pressure at cryogenic temperatures. The hybrid material showed a significant increase in hydrogen uptake compared to pristine MOF-5s and MWCNTs loaded with Pt. Compared to the earlier studies, the MOF-5 exhibited a higher uptake wt% of 1.2 and MWCNTS with 0.21 wt%. The uptake capacities of hydrogen with Pt-MWCNTs at 77 K were similar to the MWCNTs. Notably, Pt-MOFMCs adsorbed more hydrogen, *i.e.*, 1.89 wt%, than the other samples. Earlier reports on MOF-5 show that uptake capacities of hydrogen at 77 K and 1 bar pressure are between 1.2 to 1.3 wt% with a surface area of 1000 m^2^ g^−1^, indicating a spillover of hydrogen. At ambient temperature, the increase in hydrogen uptake by Pt-MOFMCs is higher than the literature reports available on the spillover mechanism. The uptake of hydrogen at 100 bar pressure by MWCNTs at 298 K is 0.20 wt%, and by MOF-5 is 0.3 wt%, which is higher than the literature data. The uptake capacities of Pt-MWCNTs (0.55 wt%) are three folds more elevated than those of pristine MWCNTs (0.2 wt%). The results revealed that in the hybrid composites (Pt-MWCNTs), the substrates and MWCNTs act as primary-spillover-receptors.^113,114^ Pt-MOFMC, which incorporates platinum multi-walled carbon nanotubes with MOF-5, has enhanced uptake capacities 4.2 times greater than MOF-5 and 2.3 times greater than Pt-MWCNTs. The secondary receptors for hydrogen uptake have not exceeded twice as the primary.^115,116^ Thus, Pt-MOFMC uptake is 2.3 times higher than Pt-MWCNT due to MOF-5 and secondary receptor porosity.

To enhance the sorption capacities of the carbon dioxide, the MOFs are incorporated with the CNTs and found that CNT doped copper BTC (CNT@Cu_3_(btc)_2_) at 298 K adsorbed 595 mg g^−1^ with 18 bar pressure compared with copper BTC alone, which is 295 mg g^−1^.^117^ In contrast, the porous carbon-based materials' uptake capacities are 164 mg g^−1^ at a pressure of 1 bar, and at 30 bar pressure, it is 1045 mg g^−1^.^118^ MOF-5@Zn_4_O(BDC)_3_ and polypropylene itaconate (PI) based novel hybrid material was prepared and characterised. The effect of increasing the weight percentage of MOF-5 to PIon on the morphology and stabilities was investigated. The increased concentration of MOF-5 in the PI structure reduced carbon dioxide adsorption capacities. The carbon dioxide uptake was high by adding MOF-5 to PI with concentrations between 1-20 wt%. However, the further increase in MOF-5 concentration beyond 40 wt% was of no use in the uptake capacities and was found to be only 65 wt%. Nevertheless, incorporating the MOF-5 into the PI decreased the composites' thermal stability more than individual compounds. The addition of MOF-5 (20 wt%) altered the film texture from smooth to coarser and denser.^119^

Testing PI for the adsorption through titration revealed that PI film alone could not adsorb the carbon dioxide. The increase in the concentration of MOF-5 to the PI enabled the film to adsorb the carbon dioxide. The incorporation of MOF-5 enhanced the composites' porosity formation and enabled the composites films (MOF-5@PI) to capture the carbon dioxide more efficiently. Vitillo *et al.* conducted similar studies on the magnesium-based MOF-5 for carbon dioxide uptake capacities^120^ and reported similar results.

Xing and Ma stated that the inter bonding of hydrogen to nitrogen (N–H⋯O and O–H⋯O) of nitrogen species enhanced the sorption capacities for carbon dioxide uptake.^121,122^ The porous carbon materials (MUCTS)with various functionalised groups and pore sizes adsorbed the carbon dioxide at 1 bar pressure in the range of 2.2 to 2.44 wt% and 3.3 to 3.7 wt% at temperatures 0° and 25 °C, respectively. However, linking with the other functionalised groups enhances carbon dioxide's sorption capacities for MUCTS. The low content of nitrogen and carboxyl groups negatively affects the absorption of carbon dioxide as the structure size does not affect the uptake. These studies revealed that the carbon dioxide uptake capacities mainly depend on the surface areas, nitrogen and carboxyl groups. Further, decreasing the temperatures from 25° to 0 °C enhanced the uptake capacities at 1 bar pressure.^123^

Besides gas separation and storage, the MOFs have applications in supercapacitors and high energy density rechargeable batteries. The mentioned characteristics of MOFs, *viz.* high surface area, appropriate functional linkers and irreplaceable morphology *etc.*, make MOFs and their composites excellent candidates for electrochemical energy storage devices. Lithium-ion batteries (LIBs) and sodium-ion batteries (SIBs) are the most imperatively explored electrochemical storage devices among supercapacitors^124,125^^.^ MOFs are the hot-spot research area in designing and constructing substantial electrode materials. MOF-5, in this scenario, successfully enhance the electrical conductivity nature and accelerate the performance of batteries in both the charging and discharging process. Zhang *et al.*^126^ have prepared mesoporous carbon material (Meso-C) using MOF-5 as the precursor. Meso-C showed excellent electrical conductivity with a high specific surface area and large pore volume. Meso-C@Se hybrid materials delivered discharge capacity up to 641 mA h g^−1^ and reversible capacity of about 306 mA h g^−1^ at a current density of 0.5C in the case of Li–Se batteries.

## 5. MOF-5 and its composites in sensing applications

The multifaceted structural MOFs display a wide range of luminescent properties. Conventionally organic and inorganic luminescent materials have been comprehensively studied and recognised for their properties in lighting, sensing, display and optical instrumentation.^127,128^ Inorganic luminescent materials *viz.* BaMgAl_10_O_17_: Eu^2+^ (blue) and GdMgB_5_O_10_: Ce^3+^, Tb^+^ (green), have been utilised as luminescent lamps. The high colour purity of rare-earth ions is the unique feature of these materials. Organic luminescent materials have been developed with applications in organic light-emitting diodes (OLEDs).^129,130^ Inorganic and organic MOFs enhance multifunctional luminescence activity as both units generate luminescence. Intermolecular interactions among organic moieties of linkers alone are weaker than those existing between metal ions (act as Lewis acids) and the organic linkers (act as Lewis bases) of MOFs, which raise the development of solid-state luminescent property of materials.^131,132^ Different guest molecules get absorbed into permanent pores of MOF materials leading to induced luminescence property by shifting wavelength. Development of MOFs with luminescence property helps design various sensing devices with detection capacity of many physical parameters like pressure, temperature, light, *etc.*^133–135^ Based on reported studies, luminescence in MOFs arises in different ways.

**Fig. 10:**
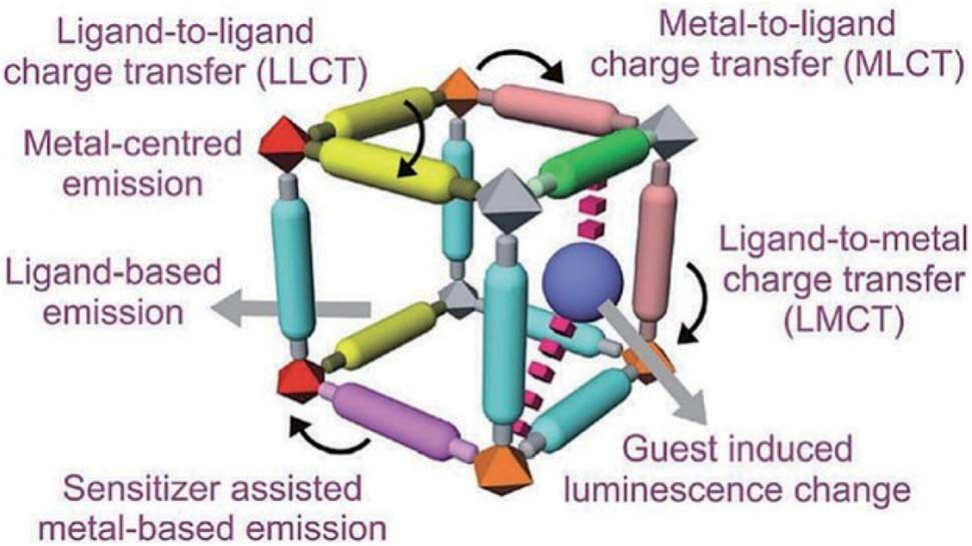
Different pathways of luminescent functionality of MOFs (this figure has been adapted/reproduced from ref. 135 with permission from the Royal Society of Chemistry, copyright 2017).

As shown in Fig. 10, along with metal and ligand, there is scope for the charge transfer from metal to the ligand and *vice versa* in MLCT and LMCT mechanisms, respectively. Furthermore, in some cases, guest molecules in MOFs also exhibit luminescence. Another added advantage of MOFs is their multifunctional behaviour. After their intended use, the substrate that causes luminescence can be used for other functionalities like magnetism, catalysis *etc.*^136–138^

Detection of carcinogenic substances is paramount, even in low concentrations. Bulky analytical instruments such as high-performance liquid chromatography (HPLC), gas chromatography-mass spectrometer (GC-MS) and thin-layer chromatography (TLC) are generally employed to detect and analyse the volatile organic compounds (VOCs). The disadvantages are laborious tasks, time-consuming and expansive instrumentation. Chemical sensors are alternative to the drawbacks. Chemical sensors are low cost, small volume and easy to use materials to detect VOCs low concentrations.^139,140^ MOF-5, in this scenario, play a prominent role in the designing of sensors.

Cobalt(ii) doped MOF-5 nanocrystals have been successfully generated using the solvothermal method to control the size and morphology. Cobalt doped MOF-5 structure remains consistent with removing temporary molecules and showing different colours by accommodating other solvent molecules in its matrix. These substances can act as solvatochromic sensing materials to identify additional solvent molecules.^141,142^

The solvatochromic performance of Co(ii)-doped MOF-5 was still present even with 7.4% cobalt content in the framework. Even bulk cobalt(ii) doped MOF sensed the solvent molecules with low speed in 25 min, attributed to high surface area and size, affecting DMF molecules' exchange rate. The detection and recognition of small molecules are independent of Co(ii) crystal morphology-doped MOF-5. The sensing capacity is through Co(ii) coordination change, *i.e.* from octahedral to a tetrahedral structure by exchanging solvent molecules. Another reason for various colours with solvent molecules' exchanges is the energy differences in d–d transitions in the visible region.^143^

In another study, CH_3_NH_3_PbBr_3_ perovskite quantum dots were introduced to the MOF-5 structure and prepared CH_3_NH_3_PbBr_3_@MOF-5 composites. Encapsulating CH_3_NH_3_PbBr_3_ in MOF-5 generates excellent sensing properties. The CH_3_NH_3_PbBr_3_@MOF-5 composite material has high thermal stability, water resistance, and flexibility compared to quantum dots. The role of metal ions on the fluorescent emission and intensity was evaluated. 4 mg of CH_3_NH_3_PbBr_3_@MOF-5 was added to 2 mL each of various aqueous solutions consisting of 1–10 M of M(NO_3_)_*x*_ (M = represents metal) subjected to 3 min sonication for uniform dispersion. The suspension's fluorescence properties depended on the type of metal ions, and sensing properties relied on the selectivity of metal ions. Aluminium, bismuth, cobalt, copper and iron ions quenched the composite's fluorescence.

Nevertheless, the aqueous solution enhanced its fluorescence intensity when the composite dispersed. The interactions of Cd^2+^ with coordinated solvent molecules and organic ligands have strengthened composite stability and thereby obtained the enhanced fluorescence intensity.^144^ This kind of MOF-5 composites advantages for developing fluorescence probes to detect various metal ions.

Abhishek *et al.*^145^ have prepared Ni-doped MOF-5 to detect NO_2_ gas. Doping with different metal ions can overcome the singular MOFs' limitations as chemical sensors and sensor devices. MOF-5 is sensitive to atmospheric conditions due to its weak metal–oxygen coordination bond and high porosity. When water molecules in the atmosphere hit the MOF-5 structure, it makes a structural change from one phase to another, eventually leading to the structure's collapse. ZnO_4_ tetrahedron moiety in the MOF-5 is the most vulnerable part for the deformation in MOF-5. Researchers have used transition metals like Ni^2+^ as substituents in the MOF matrix to circumvent this problem. Ni doping into MOF-5 at different concentrations improved its porosity, enhancing the sensing applications. NO_2_effectivelydetected through the adsorption mechanism of MOF-5 is beneficial for designing a chemical sensing device.

Pawan Kumar *et al.*^146^ have utilised the chemical sensing behaviour of MOF-5 in detecting organophosphate pesticides (OPs), which contain nitro groups. Authors have tested methyl parathion, parathion, paraoxon and fenitrothion OPs to establish MOF-5 sensing detection capability. OPs are fungicides, herbicides, and insecticide chemicals that are highly toxic and harmful to health. Sensing of OPs is essential for the detoxification process. Detection of OPs with chromatographic techniques is burdensome, demanding expensive set-ups and a skilled workforce limiting their applicability.^147^ Luminescent MOF-5 is envisaged as a useful compound for the chemosensing of OPs.The authors have incubated MOF-5 with OPs and examined the fluorescence intensity. The electrostatic interactions between electron-withdrawing –NO_2_and MOF-5 have led to a change in the emission intensity. Photoluminescence signal quenching was observed upon the interaction of MOF-5 with OPs solutions. In this study, the concentration of OPs examined in the range of 5–600 ppb and a detection limit of 5 ppb is achievable using the proposed method. This study investigated pesticides detection through quantitative enrichment. In another study, Fengming *et al.*^148^ synthesised hydrostable ZnO/MOF-5 composite with enhanced optical properties. MOF-5 is an ideal material for investigating the optical properties of MOF-semiconductor composites. ZnO is a promising semiconductor material with valuable functionalities in the design of optics and photovoltaic devices. ZnO/MOF-5 hybrid in this study has become proven material as it exhibited an enhanced photoluminescence quantum yield of 3.30% compared to bare MOF-5 (0.80%).

A few new luminescent materials co-doping Eu(iii) and Tb(iii) in MOF-5have been reported. The structure and characteristics of MOF-5 remained unaltered with doping of Eu(iii) and Tb(iii). The lanthanide-based MOFs exhibited excellent luminescent properties and are advantageous in light-emitting devices. The luminescence property of lanthanide MOFs generally accrued from trivalent lanthanide ions and organic ligands.^149,150^ Doping of different lanthanide ions in MOF-5 improves sensing efficacy. Chao *et al.*^151^ have modified MOF-5 with Eu and Tb's dopant elements. The photoluminescence study revealed the compound's potential as a probable probe for sensing temperature at a higher range from 303 to 473 K.

## 6. Conclusions

Designing and manufacturing MOFs and composites at low cost, high purity, and proper implementation scale improved practical technologies. MOF-5 is one of its kinds in the MOF family, and its combination with other materials has shown prowess as a new structural material with many potential applications. MOF-5 and its composites alleviate leaching,a significant problem with many catalysts. MOF-5 composites proved superior reusable catalysts in the C–C bond formation and other organic transformations with excellent conversion and selectivity. In photocatalysis, MOF-5 composites offer exciting opportunities to remove pollutants. MOF-5, when combined with silk fibre like entities, enhance the adsorption capacity in wastewater treatment.

On the other hand, MOF-5 composites highlight the need for careful selection to hydrogen adsorption enhancement to adopt green fuel methodologies in the automobile industry. In most cases, H_2_ uptake capacity improved in ambient conditions when MOF-5or modified MOF-5 was used as an adsorbent. The luminescent behaviour of MOF-5 and its allied materials proved capable of detecting contaminants or harmful gases at low concentrations. Despite significant developments in various fields with MOF-5, the problems persist, still with greater scope better functionalities. Modification and development are continuous processes to address challenges like moisture sensitivity, lack of mechanical resilience, and high throughput rates and improve the inherent properties to generate efficient materials with innovative applications.

## Conflicts of interest

There are no conflicts to declare.

## Supplementary Material
